# The impact of varying cluster size in cross-sectional stepped-wedge cluster randomised trials

**DOI:** 10.1186/s12874-019-0760-6

**Published:** 2019-06-14

**Authors:** James Thomas Martin, Karla Hemming, Alan Girling

**Affiliations:** Institute of Applied Health Research, College of Medical and Dental Sciences, University of Birmingham, Edgbaston, Birmingham, B15 2TT England

**Keywords:** Stepped-wedge, Cluster randomised trials, Varying cluster size

## Abstract

**Background:**

Cluster randomised trials with unequal sized clusters often have lower precision than with clusters of equal size. To allow for this, sample sizes are inflated by a modified version of the design effect for clustering. These inflation factors are valid under the assumption that randomisation is stratified by cluster size. We investigate the impact of unequal cluster size when that constraint is relaxed, with particular focus on the stepped-wedge cluster randomised trial, where this is more difficult to achieve.

**Methods:**

Assuming a multi-level mixed effect model with exchangeable correlation structure for a cross-sectional design, we use simulation methods to compare the precision for a trial with clusters of unequal size to a trial with clusters of equal size (relative efficiency). For a range of scenarios we illustrate the impact of various design features (the cluster-mean correlation – a function of the intracluster correlation and the cluster size, the number of clusters, number of randomisation sequences) on the average and distribution of the relative efficiency.

**Results:**

Simulations confirm that the average reduction in precision, due to varying cluster sizes, is smaller in a stepped-wedge trial compared to the parallel trial. However, the variance of the distribution of the relative efficiency is large; and is larger under the stepped-wedge design compared to the parallel design. This can result in large variations in actual power, depending on the allocation of clusters to sequences. Designs with larger variations in cluster sizes, smaller number of clusters and studies with smaller cluster-mean correlations (smaller cluster sizes or smaller intra-cluster correlation) are particularly at risk.

**Conclusion:**

The actual realised power in a stepped-wedge trial might be substantially higher or lower than that estimated. This is particularly important when there are a small number of clusters or the variability in cluster sizes is large. Constraining the randomisation on cluster size, where feasible, might mitigate this effect.

**Electronic supplementary material:**

The online version of this article (10.1186/s12874-019-0760-6) contains supplementary material, which is available to authorized users.

## Background

Cluster randomised trials (CRTs) often contain clusters of unequal size [4]. In the context of a parallel CRT (P-CRT), it has been established that an increase in the variability of cluster sizes leads to a decrease in precision [3, 18]. There have been many suggested modifications to the conventional cluster design effect (DE) to allow for unequal cluster sizes in a P-CRT. In such modifications, the DE is a function of the cluster sizes and the intra-cluster correlation (ICC), and either the actual (varying cluster sizes) that are known pre-trial [12, 17]; or an estimate of the average cluster size and a measure of dispersion of cluster sizes [3, 19].

**Fig. 1:**
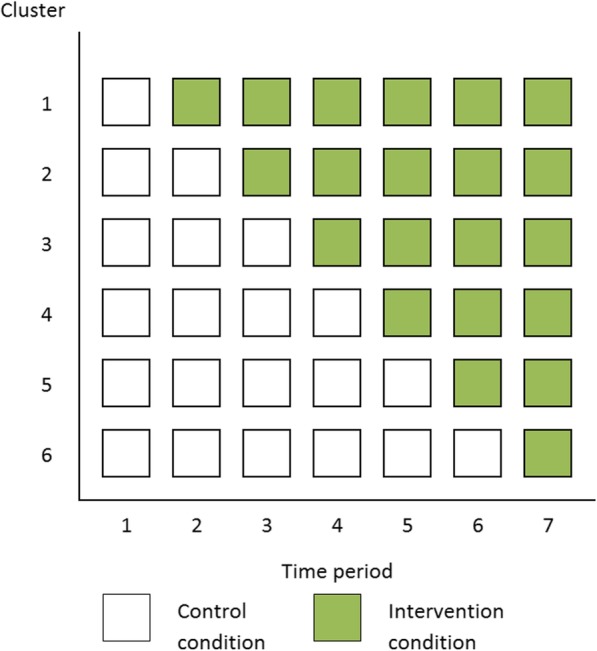
Schematic representation of a stepped-wedge cluster randomised trial with six clusters and six sequences. Each square indicates a set of observations for a given cluster at a given time-period

The stepped-wedge CRT (SW-CRT) is an alternative form of the CRT. Under this design, clusters are typically randomly allocated to one of a number of sequences which dictate how many time periods the cluster will spend in the control condition, followed by periods under the intervention condition (Fig. 1) [7, 9]. Outcomes can be assessed on the same cohort of participants who are followed-up for the study duration, on a new cross-section of participants at each time-period, or a combination of the two [2, 7]. In this paper, the focus is solely on the cross-sectional design.

Under the assumption of a cross-sectional design, [9] proposed a mixed effect model, with a fixed effect for time and random effect for cluster, as a framework for the design and analysis of a SW-CRT. They derived methods to estimate the power of a SW-CRT based on this model set up. Whilst this approach does not itself limit the cluster sizes to be equal, subsequent design effects derived from this model make the assumption that there is no variation in cluster sizes [10, 20]. Although there exists an adjustment for these DE to allow for unequal cluster sizes, it is based on stratification scheme in which the distribution of cluster sizes is the same within each sequence [6]. If the number of clusters allocated to each sequence is small, then stratification by cluster size may not be possible. Furthermore, because in a SW-CRT, clusters sequentially transition to the intervention condition, in a design with clusters of unequal size, the order of randomisation of the (different sized) clusters to cross-over to the intervention has implications on the power of the study (because it can result in a large imbalance on cluster sizes across treatment conditions). This leads to the notion of a conditional power – the power for a fixed randomisation order; and an average and distribution of power over all possible randomisation orders. At the design stage, the natural focus is on the average and distribution of power since it reflects the expected power across all randomisation orders. We therefore explore the influence of varying cluster sizes in the SW-CRTs in absence of stratification by cluster size; and importantly consider the distribution of possible realisations of power across all randomisation orders.

### Aims and objectives

We present methods to estimate the power in a SW-CRT with unequal cluster sizes in which the cluster sizes are all known, but are unequal. We then extend this method to estimate power when the cluster sizes are not known but are unequal, and only the expected average cluster size and a measure of dispersion of cluster sizes are known, such as the coefficient of variation (CV). We then explore the extent to which the power of a SW-CRT is affected by varying cluster size and highlight design variations (i.e. number of sequences, cluster size etc.) which are most influential. We illustrate how much variation in power may exist across different randomisation orders. We explore which of a SW-CRT and a P-CRT is most affected by varying cluster size; and to a limited extent explore whether randomisation schemes which constrain the randomisation so that total sample sizes under intervention and control conditions are balanced might help minimise any loss in power due to varying cluster sizes.

### Motivating example

#### Changing clinical communications: a stepped-wedge cluster randomised trial

A SW-CRT is to be used to evaluate the effectiveness of a training program aimed at improving patients’ satisfaction with doctor-patient relationship in a general practice environment. The intervention includes a training package in communication skills which will be delivered to all doctors at each of six included general practices. The intervention will be rolled-out to the practices over six sequences, and the evaluation will consist of data from seven time-periods (Fig. 1). It is unlikely that any conventional stratification method for constraining the randomisation by cluster size could be implemented in this design set up. The primary outcome is patient satisfaction score, measured via a series of questions on a Likert scale. It is hoped that the intervention will lead to a 0.2 increase in patient satisfaction from a mean (SD) patient satisfaction of 3.2 (0.8). For illustration we assume the ICC is in the region of 0.05.

Each time-period will be one month in duration, and different patients will be included at each time-period, so that the design is cross-sectional. It is expected that each cluster will contribute an average of 50 patients per time-period, so that an estimated 2100 observations (=50x6x7) will be available. However, it is known that the cluster sizes will vary. We outline two proposed approaches for accommodating this variation in cluster sizes by way of example to illustrate the concept of a distribution of power across randomisation orders, and then proceed to describe the technical details of implementation.

#### Estimating power with known cluster sizes

**Fig. 2:**
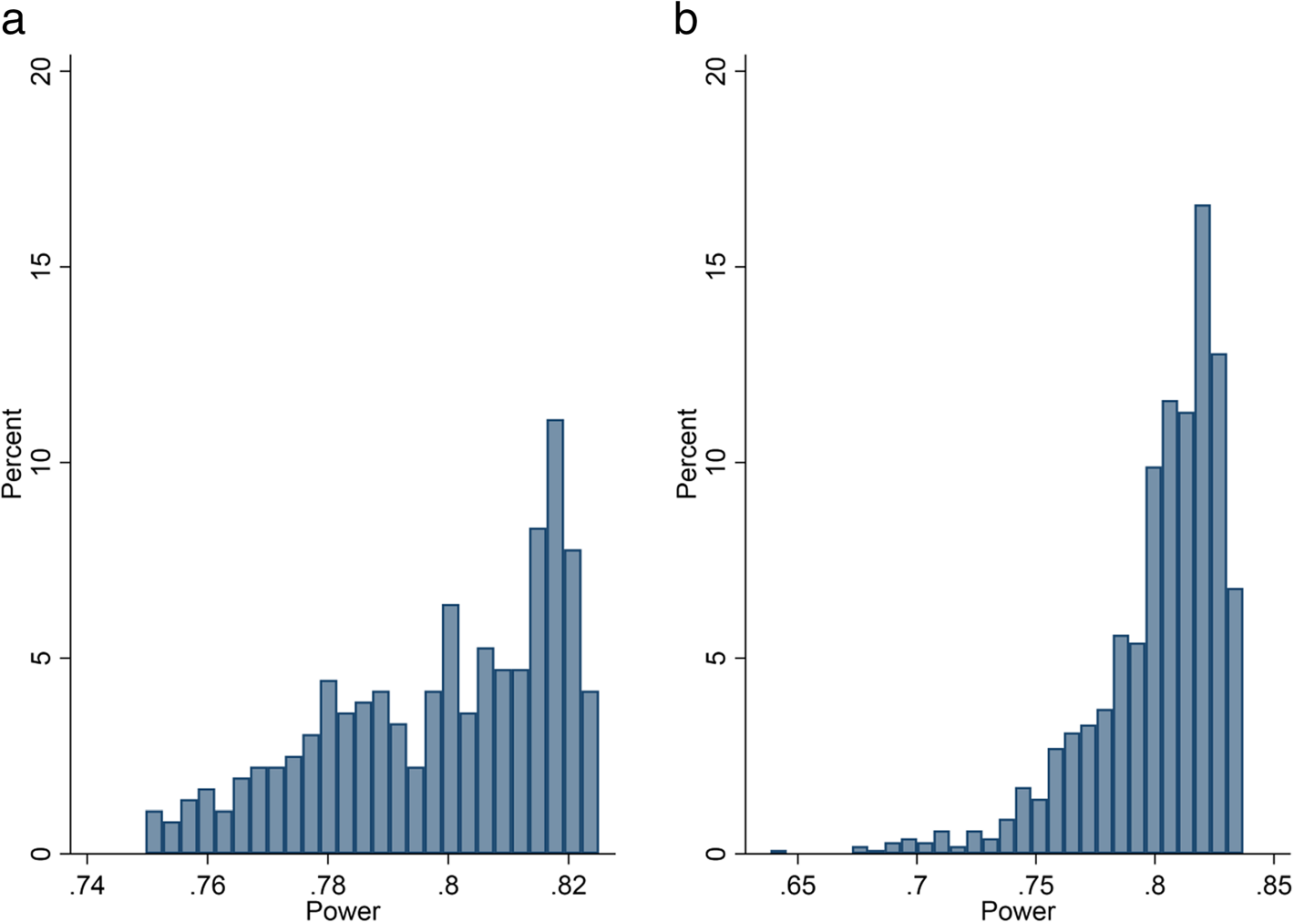
Variation in power for a cross-sectional SW-CRT with six clusters and six sequences when the cluster sizes are **a**) known and **b**) not known. **a** The clusters sizes are known pre-trial and are unequal in size – the cluster-period sizes are: 15, 25, 35, 45, 80 and 100. The power has been calculated for each possible randomisation order of the six clusters using the methods derived by Hussey and Hughes. **b** The clusters sizes are unequal but not known, but it is expected that the average cluster size per cluster-period = 50, and the coefficient of variation = 0.66. Potential cluster-period sizes have been simulated and used to estimate the power using the methods derived by Hussey and Hughes. This has been repeated 4000 times, with each simulation containing new cluster-period sizes. The SW-CRT contains six general practices randomised over six sequences, with one general practice crossing from the control to intervention condition every month. The SW-CRT is designed, such that the difference to detect = 0.2 (SD: 0.8); type 1 error = 5%

Let us first assume that the average cluster size per time-period for clusters 1 to 6 are: 15, 25, 35, 45, 80, and 100. This corresponds to an average of 50 observations per cluster-period, and a coefficient of variation of 0.66 – a value which is not dissimilar to that reported in UK general practice [3]. With six clusters and six sequences, there are 720 (=6x5x4x3x2x1) possible permutations of the randomisation order. The power can be calculated for each randomisation order using the methods described by Hussey and Hughes (described in detail forthwith), and is illustrated in Fig. 2a.

If equal cluster sizes had been assumed, then the power would be 80.75%. Allowing for the variation in cluster sizes and the associated different randomisations, the average (median) power across all randomisation orders is 80.2% (IQR: 78.4 to 81.6%, range: 75.0 to 82.5%). The minimum power is found when the randomisation order is: 25, 45, 100, 80, 15, and 35, and the maximum power when the order is: 100, 15, 45, 35, 25, and 80. Therefore, whilst on average the design may obtain 80% power, the randomisation order could produce a design in which the power is less than this, and it could be as low as 75%.

#### Estimating power with unknown cluster sizes

Let us now assume that the cluster sizes are not known, but it is known that average cluster size across clusters (per period) will be 50, and the coefficient of variation of cluster sizes will be 0.66. To acknowledge varying cluster sizes, potential (unequal) cluster sizes could be simulated, and an estimate of the power calculated using the Hussey and Hughes formula ([9], full details below). Because, the average and distribution of the possible power is of interest, the simulation of cluster sizes can be repeated a large number of times to create a distribution of the power. The distribution of power with 4000 simulated cluster-period sizes is given in Fig. 2b. The median power is 80.9% (IQR: 78.8 to 82.1%, range: 63.9 to 83.7%). Therefore, whilst on average the design may obtain 80% power, the randomisation order could produce a design in which the power is less than this, and it is estimated it could be as low as 64%.

## Methods

Firstly, for completeness, we present the method to estimate power in a SW-CRT, as presented by Hussey and Hughes [9]. This will include an illustration of how the power can be estimated for a fixed set of known, but varying, cluster sizes. We then present a simulation method to estimate the average and distribution of power across a simulated set of randomisation orders when only the mean and variance of the cluster sizes are known. We illustrate that randomisation to a particular sequence induces a conditional power and highlight the need to consider the average and distribution of power at the design stage. We then describe a simulation study that investigates the importance of variance design features (number of sequences, cluster-mean correlation, number of clusters, and coefficient of variation of cluster sizes) on the effect of the power of a SW-CRT with varying cluster size for continuous outcomes. Finally, for a limited set of scenarios we explore the correlation between the power and balance of total sample size observed under both treatment conditions.

### Estimating power in a SW-CRT with known cluster sizes (equal or unequal)

The power can be estimated in a SW-CRT using analytical methods described by Hussey and Hughes [9]. For this, a multi-level mixed effect model is assumed:

1 $$ {y}_{ij k}=\mu +{\beta}_j+{x}_{ij}\delta +{\alpha}_i+{\varepsilon}_{ij k} $$

Where, *y*_*ijk*_ is the outcome for participant *k* in cluster *i* at time *j*, *μ* is the mean outcome in the unexposed period in the first time-period, *β*_*j*_ is a time effect, fixed for time-periods*j* = 2, … , *T* (*β*_1_ = 0 for identifiability), *δ* is the treatment effect, *α*_*i*_ is a random effect for cluster *i* defined as: *α*_*i*_~*N*(0, *σ*_*b*_^2^), *ε*_*ijk*_ is the residual error (~*N*(0, *σ*_*w*_^2^)) and *x*_*ij*_ is an indicator of treatment exposure of cluster *i* at time *j* (1 = treatment, 0 = control). Under this model, the ICC can be defined as $\frac{{\sigma_b}^2}{{\sigma_b}^2+{\sigma_w}^2}$.

The power in a SW-CRT to detect a specified difference (*δ*) can be estimated using a Wald test, if the variance components are known, as:

2 $$ \phi \left(\left(\frac{\updelta}{\sqrt{{\left({X}^{\prime }{V}^{-1}X\right)}^{-1}\left[1,1\right]}}\right)-{Z}_{1-\alpha /2}\right) $$

Here, *Z*_1 − *α*/2_ is the 1 − *α*/2^*th*^ quantile of the standard Normal distribution function, *X* is a design matrix that describes the cell means for the linear parameters (the intervention effect, *δ*, the time parameters *β*_1_, … , *β*_*j*_ and the intercept *μ*) and *V* is a variance-covariance matrix of the cell means, made up of *CT* × *CT* blocks, where *C* is the number of clusters and *T* the number of time-periods. Each *T* × *T* block of *V* refers to a particular cluster and describes the correlation between the cluster means over time, and has the form:

$$ \left(\begin{array}{cccc}\frac{{\sigma_w}^2}{m_i}+{\sigma_b}^2& {\sigma_b}^2& \cdots & {\sigma_b}^2\\ {}{\sigma_b}^2& \frac{{\sigma_w}^2}{m_i}+{\sigma_b}^2& \cdots & {\sigma_b}^2\\ {}\vdots & \vdots & \ddots & \vdots \\ {}{\sigma_b}^2& {\sigma_b}^2& \cdots & \frac{{\sigma_w}^2}{m_i}+{\sigma_b}^2\end{array}\right) $$

Here *m*_*i*_ refers to the cluster-period size for cluster *i*. The *m*_*i*_ ′ *s* are known but unequal, in general.

If the cluster sizes are unequal, then the power is dependent on the randomisation order – since the randomisation will impact matrix V. A distribution of power can be calculated by considering all possible permutations of the randomisation order or a large enough sample of unique randomisation orders; and then determining the power under each of these randomisation orders.

### Estimating power in a SW-CRT with unknown (but varying) cluster sizes by simulation

In a SW-CRT in which the exact cluster sizes are not known in advance, the mean cluster-period size (*φ*) and the CV can be used to simulate potential cluster-period sizes (*m*_*i*_). Since it is expected that cluster sizes will exhibit a positive skew, and a non-negative distribution is required, we assume that the cluster-period sizes follow a Gamma distribution, such that:

$$ {m}_i\sim \Gamma \left(\alpha, \beta \right)\kern.5em E\left({m}_i\right)=\upalpha \times \beta =\varphi \kern.5em V\left({m}_i\right)={\sigma_m}^2=\alpha \times {\beta}^2={\varphi}^2\times {CV}^2 $$

The simulated values can be used in the above framework (Eq. 2) by replacing the *m*_*i*_ values in the matrix *V* in order to estimate the power. Following this, the mean cluster-period size and the CV are used to simulate a new set of cluster-period sizes. The new *m*_*i*_ values are used to calculate matrix V, which is used in Eq. 2 to calculate a new estimate of the power. This process is repeated to generate a set estimates of power, which provides an average (and distribution of) power. The number of repetitions will influence the degree of precision surrounding the mean (and possible SD) of the distribution of power.

### A simulation study to assess the impact of varying cluster size in a SW-CRT

Now, we consider the impact of various design features (such as the number of sequences and cluster-period sizes) on the power in a SW-CRT, where the cluster sizes vary. This is shown through a simulation study, which we describe below. We present estimates of the relative efficiency, which compares the precision of a SW-CRT with unequal cluster sizes to the precision in a SW-CRT with equal cluster sizes; under the prerequisite than both designs have the same total sample size. The precision is used as it is invariant to the target effect size.

**Fig. 3:**
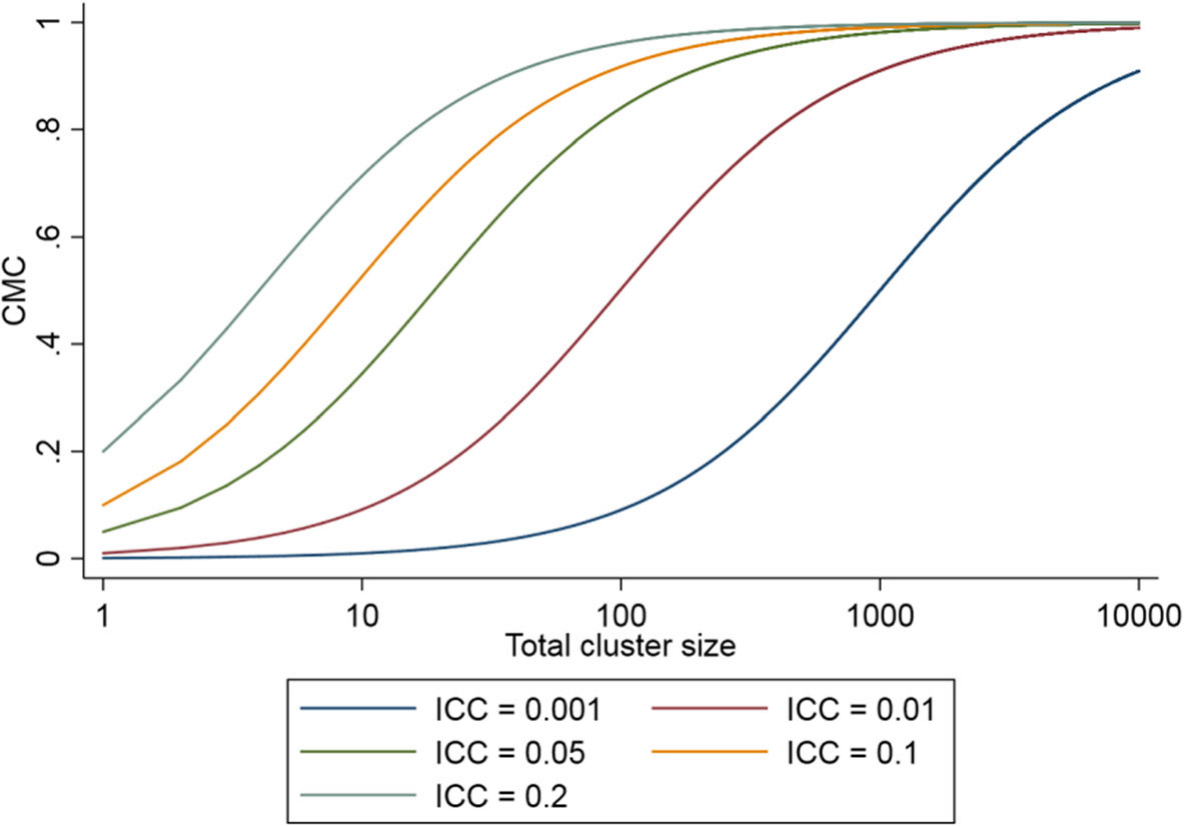
The cluster mean correlation (CMC) as a function of the total cluster size and the intracluster correlation (ICC)

We consider five key design features: the number of sequences; the number of clusters; the cluster-period size; the ICC (*ρ*); and the coefficient of variation of cluster sizes. The cluster mean correlation (CMC) is a function of the average total cluster size (M) and the ICC [5] (see Fig. 3), and represents the correlation between the cluster means of two repeated sets of observations taken from the same cluster and is defined as:

$$ R=\frac{M\times \rho }{1+\left(M-1\right)\rho } $$

It has previously been established that the efficiency of a SW-CRT with equal sized clusters hinges on the value of the CMC [5]. However, in the scenarios described below – i.e. Gamma-distributedcluster-sizes – the distribution of precision/power depends on M and *ρ* only through the CMC. This means that the number of dimensions in the simulation study is conveniently reduced by presenting results in terms of the CMC, rather than M and *ρ* separately. This has substantial presentational advantages. In what follows any result that describes the qualitative effect of an increased CMC can be re-interpreted in terms of increased *ρ*, or of increased M. The full spectrum of potential values of the CMC was used (0 to 1). The majority of SW-CRTs contain four or fewer sequences [14] but we included two larger values to capture the full effect of the number of sequences on the design and crucially because we are interested in the situation where the randomisation cannot be stratified on cluster size, which is more likely to occur in situations with a larger number of sequences. The number of clusters is based upon multiples of the number of sequences to ensure an equal number of clusters randomised per sequence. The degree of cluster size variation ranged from small (CV = 0.25) to large (CV = 1.5). A full list of the values chosen is given in Table 1. A full factorial design was used, giving 1320 possible scenarios. To maintain a Monte Carlo error around the precision smaller than 1%, 4000 simulations were used for each scenario [1].

**Table 1:** Values of the design features in a stepped-wedge cluster randomised trial and parallel cluster randomised trial that were used in the simulation study

Study design	Variable	Values used
Stepped-wedge cluster randomised trial	Number of sequences	2, 3, 4, 6, 12
	Number of clusters	12, 24, 48, 96
	Cluster mean correlation	0, 0.1, 0.2, 0.3, 0.4, 0.5, 0.6, 0.7, 0.8, 0.9, 1.0
	Coefficient of variation of cluster sizes	0.25, 0.50, 0.75, 1.00, 1.25, 1.50
Parallel cluster randomised trial	Number of clusters	12, 24, 48, 96
	Cluster mean correlation	0, 0.1, 0.2, 0.3, 0.4, 0.5, 0.6, 0.7, 0.8, 0.9, 1.0
	Coefficient of variation of cluster sizes	0.25, 0.50, 0.75, 1.00, 1.25, 1.50

Total number of possible scenarios: 1320 for stepped-wedge cluster randomised trial and 264 for parallel cluster randomised trial

In every simulation a cluster-period size (*m*_*i*_) is generated for each cluster (*i*) by sampling from a Gamma distribution with shape parameter *α*. The *m*_*i*_s are then scaled to ensure that the total sample size in the simulated design is equal to the total sample size in the corresponding equal-cluster design with cluster-period size *φ*. (The scaling ensures that the variation in simulated precision is a consequence of cluster inequality rather than differences in study-size.) The scaled *m*_*i*_s are used to calculate matrix V, which in turn is used to estimate the precision using Eq. 2, which is then compared to the precision of a SW-CRT with equal sized clusters to give the relative efficiency. This process is repeated 4000 times with a new set of cluster-period sizes simulated each time, to produce 4000 estimates of the relative efficiency. When referring to the distribution (or variation) of the relative efficiency, we focus on the IQR rather than the actual range – since the range is impacted by the number of simulations.

#### A simulation study to assess the impact of varying cluster size in a P-CRT

The notion of the randomisation of clusters impacting the precision in a SW-CRT led us consider whether the precision in a P-CRT with varying cluster sizes should also be represented as a distribution of values, rather than a singular value – which is usually assumed. The methods described above for a SW-CRT can also be used to evaluate the precision in a P-CRT [8]. This allows us to simulate potential cluster-period sizes for a P-CRT for a variety of different scenarios, and examine the impact of unequal cluster sizes in a P-CRT. The scenarios chosen were identical to that used to assess the impact of unequal cluster size in a SW-CRT (see Table 1), with the exception of the number of sequences – which can be conceptualised by two arms in a P-CRT (the total number of clusters are therefore assumed to be randomised evenly across the two arms). A full factorial design was used, giving 264 possible scenarios.

## Results

The impact of varying cluster sizes on both the average and distribution of power (or precision) of a SW-CRT depends on the design features of the study, such as the number of randomisation sequences, the CMC, and the number of clusters. We discuss the impact of each design feature in turn. The results are presented using the relative efficiency (RE), which compares the precision of a CRT with unequal cluster sizes to the precision in a CRT with equal cluster sizes; with a prerequisite that both CRTs have identical designs and sample sizes. We also discuss what impact the imbalance of observations between control and intervention conditions may have on the precision and power of a study.

### Key results

**Fig. 4:**
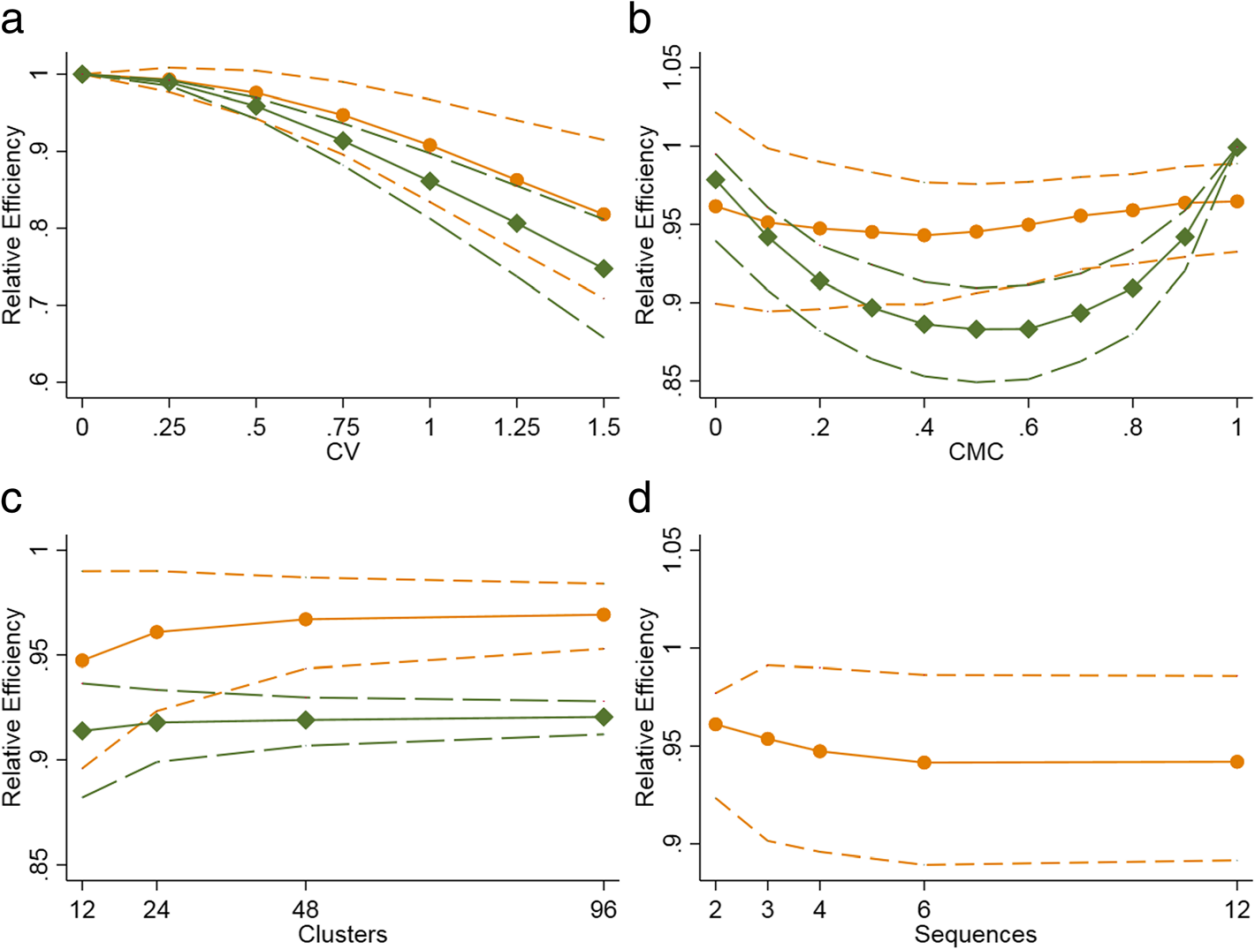
Impact of: **a**) coefficient of variation; **b**) cluster mean correlation; and **c**) number of clusters on the relative efficiency of a SW-CRT and parallel CRT, and impact of **d**) number of sequences on the relative efficiency of a SW-CRT. CV: Coefficient of variation. Efficiency is calculated as the ratio of the precision in a CRT with unequal cluster sizes compared to the precision in a CRT with equal cluster sizes. The green plots (with diamonds) are for a parallel CRT. The solid line indicates the median relative efficiency. The dashed lines indicate the lower and upper quartiles for the relative efficiency (short dashed line indicates SW-CRT). The default values for the design characteristics are (unless specified): number of clusters – 12; CV – 0.75; CMC – 0.2; number of sequences (SW-CRT only): 4

**Fig. 5:**
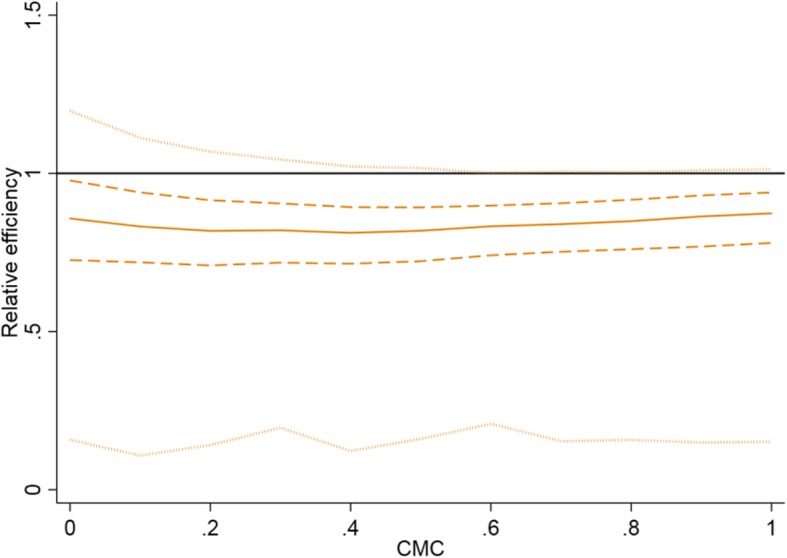
Illustrative example of the distribution of efficiency in a cross-sectional SW-CRT with 12 clusters and 4 sequences (CV = 1.5). CMC: Cluster mean correlation. Efficiency is calculated as the ratio of the precision in a SW-CRT with unequal cluster sizes compared to the precision in a SW-CRT with equal cluster sizes from 44,000 simulations (4000 simulations for each value of the cluster mean correlation). The solid line indicates the median value, the dashed line indicates the lower and upper quartiles, and the dotted line indicates the minimum and maximum values. The black solid horizontal line indicates the reference line. Above the line favours an unequal cluster size design, below the line favours an equal cluster size design

On average, the precision is lower when the cluster sizes are unequal compared to the case with equal sized clusters, for both the P-CRT and the SW-CRT (Fig. 4). Under most scenarios considered, the average effect of varying cluster sizes on precision was smaller in a SW-CRT than in a P-CRT (Fig. 4, Table 2). However, the true impact of varying cluster sizes in any given SW-CRT will depend on the randomisation of clusters to sequences. In an illustrative example, a SW-CRT with clusters of unequal size could have up to 80% less precision than a SW-CRT with equal sized clusters (Fig. 5). In the same illustrative example, somewhat surprisingly, it could transpire that a SW-CRT with clusters of unequal size could have up to 30% more precision than a SW-CRT with equal sized clusters (Fig. 5). Therefore, the anticipated precision in a SW-CRT with unequal cluster sizes might differ from a SW-CRT with equal cluster sizes, and the actual realised loss or gain in efficiency might be high and this crucially depends on the actual randomisation (i.e. there will be a range across this relative efficiency and this is not necessarily below 1).

**Table 2:** Impact of trial design on the median and inter-quartile range of possible efficiency of a SW-CRT with unequal cluster size compared so a SW-CRT with equal cluster size

CV	CMC	SW_3_		SW_12_		PD	
		12 clusters	96 clusters	12 clusters	96 clusters	12 clusters	96 clusters
0.25	0	1.00 (0.98–1.07)	1.00 (0.99–1.01)	1.00 (0.97–1.02)	1.00 (0.99–1.01)	1.00 (0.99–1.00)	1.00 (1.00–1.00)
	0.2	0.99 (0.98–1.04)	1.00 (0.99–1.00)	0.99 (0.98–1.01)	1.00 (0.99–1.00)	0.99 (0.98–0.99)	0.99 (0.99–0.99)
	0.5	0.99 (0.98–1.02)	1.00 (0.99–1.00)	0.99 (0.98–1.00)	0.99 (0.99–1.00)	0.99 (0.98–0.99)	0.98 (0.98–0.99)
	0.8	1.00 (0.99–1.01)	1.00 (1.00–1.00)	0.99 (0.99–1.00)	1.00 (1.00–1.00)	0.99 (0.99–0.99)	0.99 (0.99–0.99)
0.75	0	0.97 (0.90–1.02	1.00 (0.98–1.01)	0.96 (0.90–1.02)	0.99 (0.97–1.02)	0.98 [0.94–1.00)	1.00 (0.99–1.00]
	0.2	0.95 (0.90–0.99)	0.97 (0.96–0.98)	0.94 (0.89–0.99)	0.96 (0.95–0.98)	0.91 (0.88–0.94)	0.92 (0.91–0.93]
	0.5	0.95 (0.91–0.98)	0.97 (0.96–0.98)	0.94 (0.90–0.97)	0.96 (0.95–0.97)	0.88 (0.85–0.91)	0.88 (0.87–9.90)
	0.8	0.96 (0.92–0.99)	0.99 (0.98–0.99)	0.96 (0.93–0.97)	0.98 (0.98–0.99)	0.90 (0.88–0.93)	0.93 (0.89–0.91)
1.25	0	0.91 (0.79–1.00)	1.00 (0.95–1.02)	0.89 (0.78–0.99)	0.98 (0.94–1.02)	0.94 (0.82–0.99)	0.99 (0.98–1.00)
	0.2	0.88 (0.78–0.95)	0.93 (0.91–0.95)	0.85 (0.76–0.93)	0.92 (0.90–0.94)	0.81 (0.74–0.86)	0.82 (0.80–0.83)
	0.5	0.88 (0.79–0.93)	0.93 (0.91–0.95)	0.85 (0.79–0.90)	0.91 (0.90–0.92)	0.73 (0.67–0.78)	0.73 (0.71–0.75)
	0.8	0.90 (0.81–0.95)	0.96 (0.95–0.98)	0.88 (0.83–0.92)	0.95 (0.94–0.96)	0.76 (0.70–0.81)	0.75 (0.73–0.77)

*CMC* Cluster mean correlation, *CV* coefficient of variation in cluster sizes, *SW*_*3*_ Stepped-wedge trial with 3 sequences, *SW*_*12*_ Stepped-wedge trial with 12 sequences (one cluster per sequence), *PD* Parallel design

The magnitude of the loss or gain in efficiency and its possible range across randomisation orders is impacted by the design features of the SW-CRT, which are discussed in more detail below. We focus on the inter-quartile range so as not to put undue emphasis on extremes.

### Stepped-wedge CRTs

#### Coefficient of variation of cluster sizes

Any increase to the amount of variation in cluster sizes leads to a greater average precision loss in a SW-CRT (i.e. the RE is less than one). Figure 4a illustrates a small amount of variability in cluster sizes (CV = 0.25) has negligible impact on the average RE, but larger amounts of cluster size variability could provide a design with substantial losses in efficiency compared to a design with equal sized clusters. In addition, the range of the distribution of RE values widens as the CV increases. For example a 12-clusterSW-CRT with 12 sequences and a CMC of 0.2, the RE has an IQR of 0.98 to 1.02 when the CV is small (CV = 0.25) (Table 2); whereas there is a much wider IQR of 0.76 to 0.93 when the CV is large (CV = 1.25).

#### Cluster mean correlation

The average loss in precision in a SW-CRT due to the presence of unequal sized clusters is relatively unaffected by the CMC (Fig. 4b). However, the actual value of the RE can vary substantially from the average depending on the randomisation of clusters to sequences. Figure 4b illustrates how the range (or distribution) of the RE is widest when the CMC is small; and at its narrowest when the CMC is large. This is emphasised by an example from Table 2, in which for a SW-CRT with 3 sequences and 12 clusters the average RE is 0.91 [IQR: 0.79–1.00] for a CMC of 0; and 0.90 [IQR: 0.81–0.95] for a CMC of 0.8 (illustrative CV = 1.25).

#### Number of clusters

The average loss in efficiency due to unequal sized clusters does depend on the number of clusters and is greater when the number of clusters is small (Fig. 4c). The range of the RE also depends on the number of clusters. Figure 4c illustrates that the range of the RE is widest when the number of clusters is smaller; and at its narrowest when the number of clusters is large. For example, the average RE for the 12 sequence design is 0.88 [IQR: 0.83–0.92] for a study with 12 clusters (Table 2); and 0.95 [IQR: 0.94–0.96] for a study with 96 clusters (illustrative CV = 1.25 and CMC = 0.8).

#### Number of sequences

The average loss in precision due to the presence of unequal sized clusters is relatively unaffected by the number of sequences. The range (or distribution) of the RE is not greatly impacted by the number of sequences when the SW-CRT has more than two sequences (Fig. 4d). For example from Table 2, in a SW-CRT with 12 clusters the average RE for a design with 3 sequences is 0.90 [IQR: 0.81–0.95]; and 0.88 [IQR: 0.83–0.92] for a design with 12 sequences (illustrative CV = 1.25 and CMC = 0).

##### SW-CRT vs P-CRT

The effect of varying cluster sizes on the average loss (or gain) in efficiency is smaller in a SW-CRT compared to the P-CRT (Fig. 4a, c, Table 2). However, as is the case for the SW-CRT, the actual realised precision (or power) in a P-CRT might be different from the expected (or average) precision. The relationship between the average and distribution of precision, and the number of clusters and amount of variation in cluster sizes (CV) is similar to that of the SW-CRT. Any increase in the CV leads to a decrease in the average RE, and a widening of the range of RE values. P-CRTs with fewer clusters may have a lower RE and a wider range of RE values than designs with a greater number of clusters. However, the relationship between the relative efficiency and the cluster-mean correlation in a P-CRT is somewhat different to in a SW-CRT (Fig. 4b). As previously discussed, the impact of the CMC in a SW-CRT is small. However in a P-CRT, increases to the CMC between 0 and 0.5 lead to decreases in the RE, but increases in the CMC between 0.5 and 1.0 increase the RE, and so the P-CRT follows a parabolic pattern when comparing RE and the CMC. Furthermore, in a SW-CRT it is possible for designs with unequal cluster sizes to obtain more precision – and hence a greater power – than an identical SW-CRT but with equal sized clusters. However, a P-CRT with unequal cluster sizes can never have greater precision than a P-CRT with equal sized clusters (Fig. 4a, c, Table 2).

##### Imbalance of observations between control and intervention condition (sample size imbalance)

**Fig. 6:**
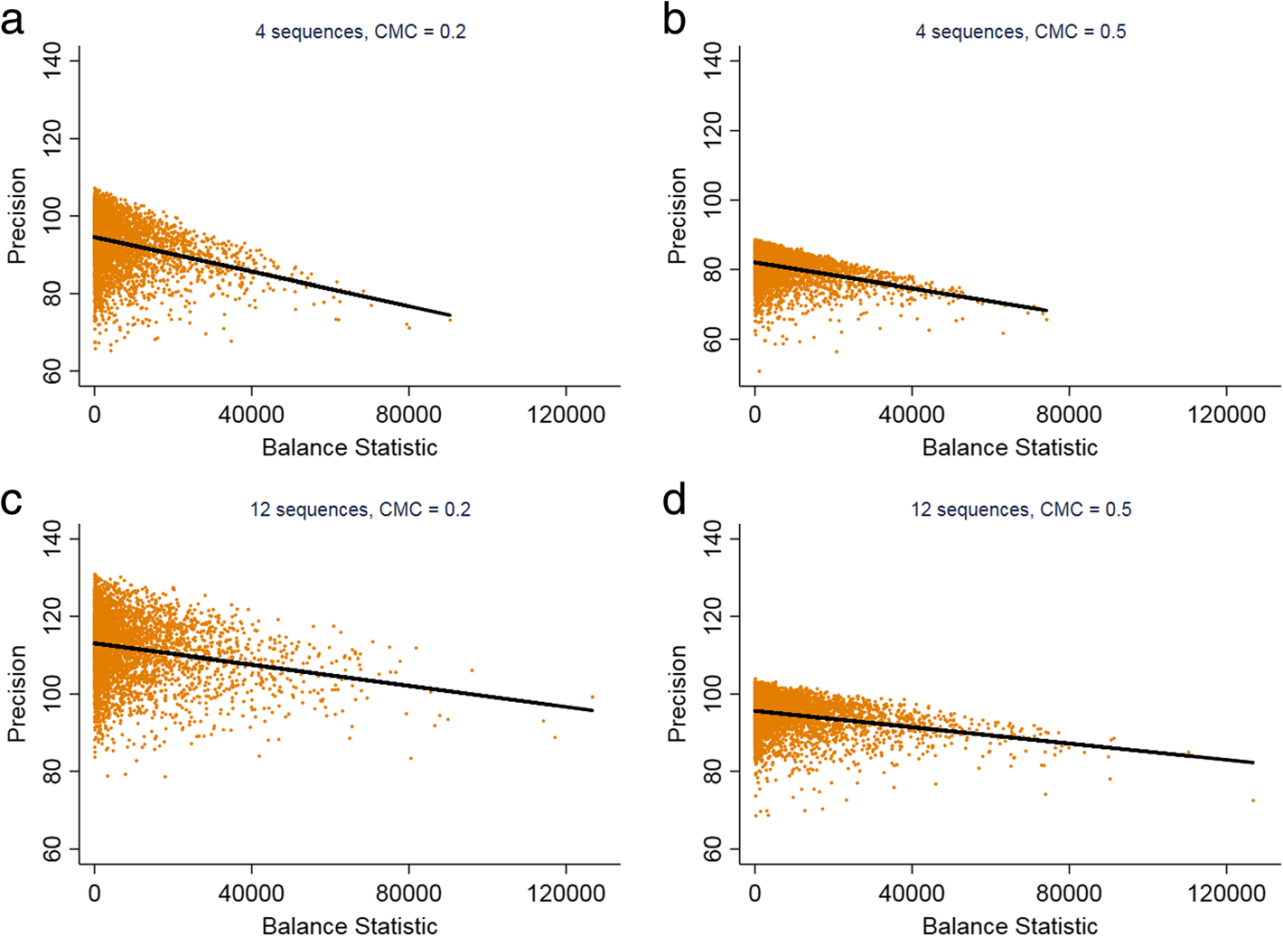
The impact of imbalance of observations between control and intervention condition on the precision of a stepped-wedge cluster randomised trial. CMC: Cluster mean correlation. The balance statistic was calculated as: (number of observation in intervention condition – number of observation in control condition)2. A larger value of the balance statistic indicates greater imbalance. Each point is the balance statistic and power for a particular set of simulated cluster sizes. The default values for the design characteristics are: number of clusters: 12; CV: 1.5

In a SW-CRT with clusters of unequal sizes, the randomisation process could lead to an imbalance in the number of observations contributing to the control and intervention conditions (sample size imbalance). However, the guarantee of an equal number of observations observed under control and intervention conditions does not guarantee that a SW-CRT will have optimal precision or power. A comparison of precision and sample size imbalance has been illustrated for four scenarios in Fig. 6 (12 cluster SW-CRT with 4 or 12 sequences and a CMC of 0.2 or 0.5). Generally, the lowest precision was found when there was a small degree of sample size imbalance. The greatest precision is not necessarily achieved when the number of observations is equal in control and intervention conditions. The results are consistent with changes to the number of sequences and changes to the CMC. However, despite these scenarios showing a positive correlation between sample size imbalance and precision, in several other examples (a SW-CRT with 4 clusters and 4 sequences, and a SW-CRT with 5 clusters and 5 sequences), we observed the opposite relationship (see Additional file 1: Figure S1).

## Discussion

It is well known that the precision or power of a cluster randomised trial is lower when the cluster sizes are unequal compared to the case with equal sized clusters. This is known to be the case for both the P-CRT and the SW-CRT. More recently, it has also been established that the average reduction in relative precision in a SW-CRT is lower than in a P-CRT [6]. However, we have shown that whilst the expected or average impact of varying cluster sizes is relatively small, the actual impact might be much larger. This is because conditional on the randomisation order, a SW-CRT with clusters of unequal size could possibly have more or less precision than a SW-CRT with equal sized clusters. In some designs with unequal cluster sizes, some randomisations could lead to as much as a 30% increase in precision compared to a design with equal sized clusters. However, other randomisations could lead to an 80% decrease in precision compared to a design with clusters of equal size. These potentially large reductions (or sometimes increases) in precision are particularly of concern in SW-CRTs with large variation between cluster sizes, a small number of clusters or small cluster-mean correlation (i.e. smaller cluster sizes or smaller intra-cluster correlation).

We also demonstrated similar, although less marked properties in the P-CRT. This is something that has not been noted in the literature to date. In the P-CRT it has been established that the loss of (average) efficiency due to variation in cluster sizes rarely exceeds 10% [6, 19]. However, this average or expected loss in efficiency holds under the assumption of a size-stratified randomisation scheme [6]. When not stratifying the randomisation on cluster size, the loss of efficiency can greatly exceed 10% depending on the randomisation order. It is fairly typical for a P-CRT to stratify or constrain the randomisation on cluster size [11]. A constrained randomisation approach has been recommended to minimise loss in power in a SW-CRT [16]. We observed (for a limited set of scenarios) that on average, the smaller the sample size imbalance, the greater the precision. However, for a few limited scenarios, we observed an inverse correlation between sample size imbalance and precision. Further work is therefore needed to determine when a constrained randomisation in SW-CRTs, where the constraint minimises any sample size imbalance, will achieve desired aims of increasing power and where it might decrease power.

### Limitations

Although the methods and results described here have been for continuous outcomes, we suggest that until further research is conducted these results are also assumed to hold for binary outcomes. In this work, we have assumed an exchangeable correlation structure with only random cluster effects. Further work is needed to consider more general auto-correlation structures. For example, the inclusion of a random cluster by time interaction [6, 10, 15], or an exponential correlation model [13]. We also assumed observations were sampled uniformly across time-periods, which is consistent with standard approaches for longitudinal cluster randomised trials, but may not always be an assumption that will hold in practice.

### Conclusions

The actual realised power in a stepped-wedge trial with unequal cluster sizes depends on the order of randomisation of clusters to sequences. Design inflation factors, allowing for varying cluster sizes, all assume a size-stratified randomisation scheme. Only under this assumption is the impact of varying cluster size known to be minimal. Where randomisation schemes either do not, or where it is infeasible to implement a size-stratified randomisation scheme, the realised power could be substantially higher or lower than the expected power, even after allowing for variation in cluster sizes. This is particularly important when there are a small number of clusters or the variability in cluster sizes is large. Constraining the randomisation on cluster size, where feasible, might mitigate this effect.

## Additional file

Additional file 1: **Figure S1.** The impact of imbalance of observations between control and intervention condition on the precision of a stepped-wedge cluster randomised trial with few clusters. The balance statistic was calculated as: (number of observation in intervention condition – number of observation in control condition). A larger value of the balance statistic indicates greater imbalance. Each point is the balance statistic and precision for a particular randomisation order. Values were calculated for all possible randomisation orders. The cluster sizes for the 4 cluster design (a) are: 10, 50, 100, and 500. The cluster sizes for the 5 cluster design (b) are: 15, 25, 50, 100, and 200. (PNG 40 kb)

## Data Availability

The datasets used and analysed during the current study are available from the corresponding author on reasonable request.

## Abbreviations

CMC: Cluster mean correlation
CRT: Cluster randomised trial
CV: Coefficient of variation
DE: Design effect
ICC: Intracluster correlation
P-CRT: Parallel cluster randomised trial
SW-CRT: Stepped-wedge cluster randomised trial

## References

[1] Baio G, Copas A, Ambler G, Hargreaves J, Beard E, Omar RZ (2015). Sample size calculation for a stepped wedge trial. Trials..

[2] Copas AJ, Lewis JJ, Thompson JA, Davey C, Baio G, Hargreaves JR (2015). Designing a stepped wedge trial: three main designs, carry-over effects and randomisation approaches. Trials..

[3] Eldridge SM, Ashby D, Kerry S (2006). Sample size for cluster randomized trials: effect of coefficient of variation of cluster size and analysis method. Int J Epidemiol.

[4] Eldridge SM, Ashby D, Feder GS, Rudnicka AR, Ukoumunne OC (2004). Lessons for cluster randomized trials in the twenty-first century: a systematic review of trials in primary care. Clinical trials.

[5] Girling AJ, Hemming K (2016). Statistical efficiency and optimal design for stepped cluster studies under linear mixed effects models. Stat Med.

[6] Girling A. Relative efficiency of unequal cluster sizes in stepped wedge and other trial designs under longitudinal or cross-sectional sampling. Stat Med. 2018:1–13. 10.1002/sim.7943 PMC6635744 30209812

[7] Hemming K, Haines TP, Chilton PJ, Girling AJ, Lilford RJ (2015). The stepped wedge cluster randomised trial: rationale, design, analysis, and reporting. BMJ (Clinical research ed)..

[8] Hemming K, Lilford R, Girling AJ (2015). Stepped-wedge cluster randomised controlled trials: a generic framework including parallel and multiple-level designs. Stat Med.

[9] Hussey MA, Hughes JP (2007). Design and analysis of stepped wedge cluster randomized trials. Contemporary clinical trials.

[10] Hooper R, Teeresntra S, De Hoop E, Eldridge S (2016). Sample size calculations for stepped wedge and other longitudinal cluster randomised trials.

[11] Ivers NM, Halperin IJ, Barnsley J, Grimshaw JM, Shah BR, Tu K (2012). Allocation techniques for balance at baseline in cluster randomized trials: a methodological review. Trials..

[12] Kerry SM, Bland JM (2001). Unequal cluster sizes for trials in English and welsh general practice: implications for sample size calculations. Stat Med.

[13] Kasza J, Hemming K, Hooper R, Matthews J, Forbes AB. Kasza J, Hemming K, Hooper R, Matthews J, Forbes AB; ANZICS Centre for Outcomes & Resource Evaluation (CORE) Committee. Impact of non-uniform correlation structure on sample size and power in multiple-period cluster randomised trials. Stat Methods Med Res. 2017. 1:962280217734981. 10.1177/0962280217734981 29027505

[14] Martin J, Taljaard M, Girling AJ, Hemming K (2016). Systematic review finds major deficiencies in sample size methodology and reporting for stepped-wedge cluster randomised trials. BMJ Open.

[15] Martin J, Girling A, Nirantharakumar K, Ryan R, Marshall T, Hemming K (2016). Intra-cluster and inter-period correlation coefficient for cross-sectional cluster randomised controlled trials for type-2 diabetes in UK primary care. Trials..

[16] Moulton LH, Golub JE, Durovni B, Cavalcante SC, Pacheco AG, Saraceni V, King B, Chaisson RE (2007). Statistical design of THRio: a phased implementation clinic-randomized study of a tuberculosis preventive therapy intervention. Clin Trials.

[17] Pan W (2001). Sample size and power calculations for correlated binary data. Control Clin Trials.

[18] Rutterford C, Copas A, Eldridge S (2015). Methods for sample size determination in cluster randomized trials. Int J Epidemiol.

[19] Van Breukelen GJ, Candel MJ (2012). Comments on ‘efficiency loss because of varying cluster size in cluster randomized trials is smaller than literature suggests. Stat Med.

[20] Woertman W (2013). Stepped wedge designs could reduce the required sample size in cluster randomized trials. J Clin Epidemiol.

