# Supplementary figures and images for: The impact of varying cluster size in cross-sectional stepped-wedge cluster randomised trials
Source: BMC Med Res Methodol. 2019 Jun 14;19:123. doi: 10.1186/s12874-019-0760-6 (PMC6570871; doi:10.1186/s12874-019-0760-6)

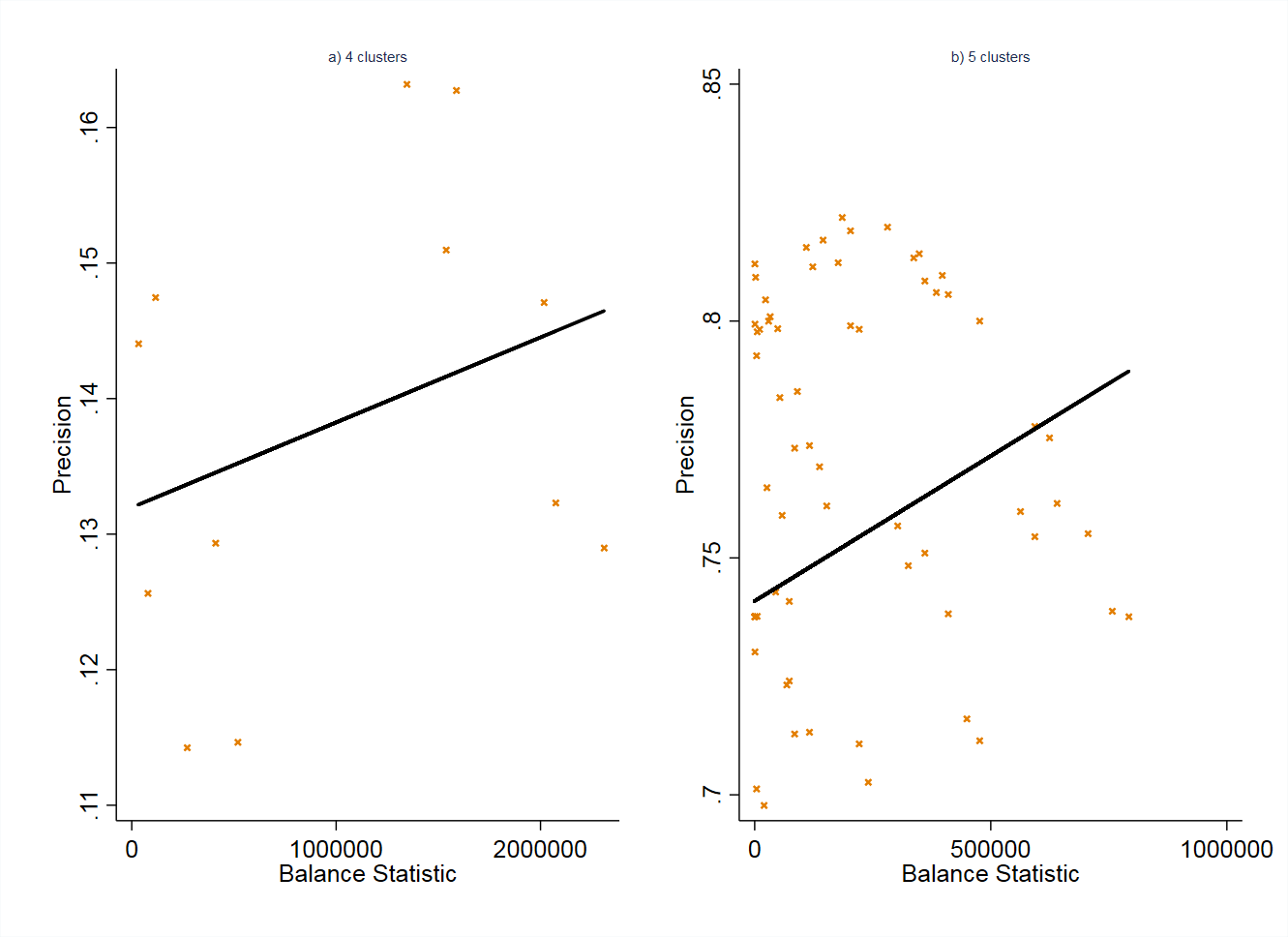

Supplement: Supplementary file 1 — Figure S1. The impact of imbalance of observations between control and intervention condition on the precision of a stepped-wedge cluster randomised trial with few clusters. The balance statistic was calculated as: (number of observation in intervention condition – number of observation in control condition). A larger value of the balance statistic indicates greater imbalance. Each point is the balance statistic and precision for a particular randomisation order. Values were calculated for all possible randomisation orders. The cluster sizes for the 4 cluster design (a) are: 10, 50, 100, and 500. The cluster sizes for the 5 cluster design (b) are: 15, 25, 50, 100, and 200. (PNG 40 kb) [file 12874_2019_760_MOESM1_ESM.png]
